# Survival of Gastric Cancer Patients at a Tertiary Care Hospital in Eastern India: A Retrospective Data Analysis

**DOI:** 10.7759/cureus.37064

**Published:** 2023-04-03

**Authors:** Smruti Priyambada Pradhan, Sunil Agarwala, Jyotiranjan Sahoo, Sukant Kumar Pradhan, Shubharanjan Jena, Nancy Satpathy, Venkatarao Epari

**Affiliations:** 1 Community Medicine, Siksha 'O' Anusandhan Deemed to be University Institute of Medical Sciences and SUM Hospital, Bhubaneswar, IND; 2 Surgical Oncology, Siksha 'O' Anusandhan Deemed to be University Institute of Medical Sciences and SUM Hospital, Bhubaneswar, IND; 3 Bioinformatics, Odisha University of Agriculture and Technology, Bhubaneswar, IND

**Keywords:** clinicopathological, sociodemographic, mortality, log-rank test, kaplan-meier method

## Abstract

Background

Gastric cancer is one of the most common cancers and a leading cause of death worldwide. Most cases of gastric cancer are diagnosed at an advanced stage when no definitive treatment is available leading to an overall declined survival rate. In this study, we aimed to investigate the survival rate of gastric cancer patients admitted to our tertiary care center and determined the relationship between sociodemographic and clinicopathological characteristics with mortality.

Methodology

Gastric cancer patients treated between January 2019 and December 2020 were included in this retrospective study. The clinicopathological and demographic data of 275 gastric cancer patients were analyzed. The Kaplan-Meier method was used to calculate the overall survival of gastric cancer patients. The Kaplan-Meier log-rank test was used to calculate the difference.

Results

The mean survival of gastric cancer patients was 20.10 months (95% confidence interval = 19.20-21.03). Deaths were higher among stage III (42.6%) and stage IV (36.1%) patients compared to stage I (1.6%) and stage II (19.7%) patients. Mortality was significantly higher (70.5%) in patients without surgery.

Conclusions

The mean survival in our study setting is lower and is associated with the pathological stage of the disease, surgical intervention, and patients presenting with other gastrointestinal symptoms. A lower survival rate can be attributed to late diagnosis.

## Introduction

Cancer is the second leading cause of death worldwide causing premature death among people aged 30-69 years, with an estimated 18.1 million new cancer diagnoses in 2018 alone [1,2]. The cancer burden is increasing unequally among the populations of low- and middle-income countries [2].

Gastric cancer ranks fifth in terms of incidence and third in terms of mortality [3,4]. An estimated 1 million new gastric cancer cases were diagnosed and 0.78 million deaths occurred in 2018 [1]. Among the poverty-related non-communicable diseases, infection-related cancers including gastric cancer are an additional burden among lower-income countries [1].

India with a population of 1.35 billion has reported 1.16 million new cancers and 0.78 million cancer deaths. In addition, one in 15 Indians is at risk of dying due to cancer during their lifetime, while one in 10 Indians is at risk of developing new cancer [2]. Gastric cancer (approximate incidence of 39,000) is one of the six most common cancers reported in 2018 [1].

Most studies indicate that gastric cancer is diagnosed in old age and the majority of patients are males [5,6]. The survival rate among the older age group is affected by the difficulties inherent in their care. The majority of individuals are diagnosed at advanced stages when standard treatment options such as surgery are ineffective. Thus, the survival rate is reduced [7]. The survival rate of gastric cancer is very low at less than 20% [8], especially among individuals of low socioeconomic strata [1]. Gastric cancer survival depends on various circumstances, including the stage of the disease, the patient’s primary complaint, the type of treatment, and people’s lifestyles, cultures, and food habits [9].

Relatively scanty literature has explored gastric cancer survival and its associated factors, especially in this region. It is important to have regional information about gastric cancer survival to generate baseline information and guide further management of gastric cancer. This study aimed to determine the overall survival rate of gastric cancer patients admitted to our tertiary care center and find an association of mortality with sociodemographic and clinicopathological factors.

## Materials and methods

Study setting

Institute of Medical Sciences and SUM Hospital, Odisha is one of the premier medical colleges and hospitals providing oncology services in all three divisions (namely, surgical, radiotherapy, and chemotherapy) including pediatric oncology. It has a daily footfall (old and new cases) of more than 3,000 patients and approximately 30 surgical oncology cases.

Sample size/sampling

Because all eligible patients were included in the study, sampling/sample size calculation was not done. This was a retrospective record review and the follow-up data mentioned in the case sheets were retrieved for data analysis.

Data collection

In this retrospective study, we included gastric cancer patients who were treated and followed up at our tertiary care center for two years from January 2019 to December 2020. We only included biopsy-confirmed gastric cancer patients irrespective of age and gender. Reviewing the medical records, we found 311 patients satisfying our inclusion criteria. Those with incomplete data pertaining to the objectives (n = 18), those with incomplete follow-up (n = 12), and those having other associated malignancies were excluded (n = 6). Data on age, gender, family history (first-degree relatives) of gastric cancer, smoking history, pathological stage (TNM classification), type, and survival status were collected retrospectively.

The domiciliary status of the patient was ascertained from the district of origin, and the districts were classified into central, northern, and southern districts according to the local government.

Statistical analysis

Continuous variables were expressed as mean ± standard deviation (SD) and categorical variables as number and percentage. The chi-square test was used to find an association between categorical variables, and the Student’s t-test was used to compare the means. The Kaplan-Meier method was used to calculate the overall survival of gastric cancer patients, and a log-rank test was used to calculate the difference. Statistical analysis was carried out using SPSS version 27.0 (IBM Corp., Armonk, NY, USA) software licensed to the institute.

Ethical consideration

No primary data were collected from the study patients. However, patient identifiers were concealed from publication. Prior ethical approval was obtained from the institutional ethics committee (ref.no/DRI/IMS.SH/SOA/2021/076 dated June 21, 2021).

## Results

A total of 275 gastric cancer patients were included in this study. The majority (94.18%) had adenocarcinoma and the remaining had signet ring cell and gastrointestinal stromal tumors. Among them, 60.7% were males with a mean age of 59 ± 12.83 years, whereas the mean age of females (39.3%) was 54.01 ± 13.41 years. Gastric cancer patients had come from all 30 districts of Odisha. We did not find any statistically significant association of mortality with sociodemographic and personal characteristics of gastric cancer patients (Table 1).

**Table 1:** Association of mortality with sociodemographic and personal characteristics of gastric cancer patients.

Variable	Alive n (%)	Died n (%)	Total n (%)	P-value
Gender				
Male	133 (62.1)	34 (55.7)	167 (60.7)	0.366
Female	81 (37.9)	27 (44.3)	108 (39.3)	
Age				
Mean age ± SD	54.13 ± 12.838	53.38 ± 13.341		0.691
Median age ± IQR	55 ± 16	55 ± 15.5		
All forms of tobacco				
Yes	89 (41.6)	23 (37.7)	112 (40.7)	0.586
No	125 (58.4%)	38 (62.3%)	163 (59.3%)	
Smoking				
Yes	53 (24.8)	10 (16.4)	63 (22.9)	0.170
No	161 (75.2%)	51 (83.6%)	212 (77.1%)	
Family history				
Yes	46 (21.5)	13 (21.3)	59 (21.50)	0.975
No	168 (78.5)	48 (78.7)	216 (78.50)	
District				
Central	134 (62.6)	43 (70.5)	177 (64.4)	0.508
Northern	43 (20.1)	9 (14.8)	52 (18.9)	
Southern	37 (17.3)	9 (14.8)	46 (16.7)	

The median follow-up time was 15 months. A significantly higher proportion of stage III (42.6%) and stage IV (36.1%) patients died compared to stage I (1.6%) and stage II (19.7%) patients (p = 0.007). Mortality was significantly higher (70.5%) in patients who had not undergone surgery (p = 0.006). We did not find any significant association of mortality with the histopathologic type and other forms of treatment (Table 2).

**Table 2:** Association of histopathology and treatment history with mortality due to gastric cancer.

Variable	Alive n (%)	Death n (%)	Total n (%)	P-value
Stage				
I	23 (10.7)	1 (1.6)	24 (8.7)	0.007
II	73 (34.1)	12 (19.7)	85 (30.9)	
III	62 (29.0)	26 (42.6)	88 (32.0)	
IV	56 (26.2)	22 (36.1)	78 (28.4)	
Histopathological type				
Adenocarcinoma	199 (93.0)	60 (98.4)	259 (94.2)	0.114
Others	15 (7.0)	1 (1.6)	16 (5.8)	
Surgery				
Yes	106 (49.5)	18 (29.5)	124 (45.1)	0.006
No	108 (50.5)	43 (70.5)	151 (54.9)	
Chemotherapy				
Yes	94 (43.9)	20 (32.8)	114 (41.5)	0.119
No	120 (56.1)	41 (67.2)	161 (58.5)	
Radiotherapy				
Yes	3 (104)	0 (0)	3 (1.1)	0.998
No	211 (98.6)	61 (100)	272 (98.9)	
Combination treatment				
Yes	2 (0.9)	0 (0)	2 (0.7)	0.998
No	212 (99.1)	61 (100)	273 (99.3)	

Gastrointestinal (GI) symptoms such as abdominal pain, melena, hematemesis, dysphagia, and anorexia were the predominant patient complaints. These symptoms were almost equally distributed among patients and did not have a significant association with mortality. Similarly, symptoms such as weight loss, generalized weakness, and neurological symptoms were not associated with mortality. However, other GI symptoms such as nausea, ulcer, and hiccups were higher among survivors of gastric cancer (p = 0.043) (Table 3).

**Table 3:** Association of clinical symptoms with mortality due to gastric cancer.

Variable	Alive n (%)	Death n (%)	Total n (%)	P-value
Presented with complaint				
Yes	194 (90.7)	55 (90.2)	249 (90.50)	0.908
No	20 (9.3)	6 (9.8)	26 (9.50)	
Abdominal pain				
Yes	79 (36.9)	22 (36.1)	101 (36.7)	0.903
No	135 (63.1)	39 (63.9)	174 (63.3)	
Malena				
Yes	54 (25.2)	11 (18.0)	65 (23.6)	0.243
No	160 (74.8)	50 (82.0)	210 (76.4)	
Hematemesis				
Yes	50 (23.4)	16 (26.2)	66 (24.0)	0.644
No	164 (76.6)	45 (73.8)	209 (76.0)	
Anorexia				
Yes	33 (15.4)	13 (21.3)	46 (16.7)	0.277
No	181 (84.6)	48 (78.7)	229 (83.3)	
Dysphagia				
Yes	60 (28.0)	23 (37.7)	83 (30.2)	0.147
No	154 (72.0)	38 (62.3)	192 (69.8)	
Weight loss				
Yes	15 (7.0)	6 (9.8)	21 (7.6)	0.463
No	199 (93.0)	55 (90.2)	254 (92.4)	
General weakness				
Yes	17 (7.9)	5 (8.2)	22 (8.00)	0.949
No	197 (92.1)	56 (91.8)	253 (92.0)	
Neurological problems				
Yes	6 (2.8)	3 (4.9)	9 (3.3)	0.413
No	208 (97.2)	58 (95.1)	266 (96.7)	
Other gastrointestinal problems				
Yes	88 (41.1)	34 (55.7)	122 (44.4)	0.043
No	126 (58.9)	27 (44.3)	153 (55.6)	

Survival analysis

**Figure 1:**
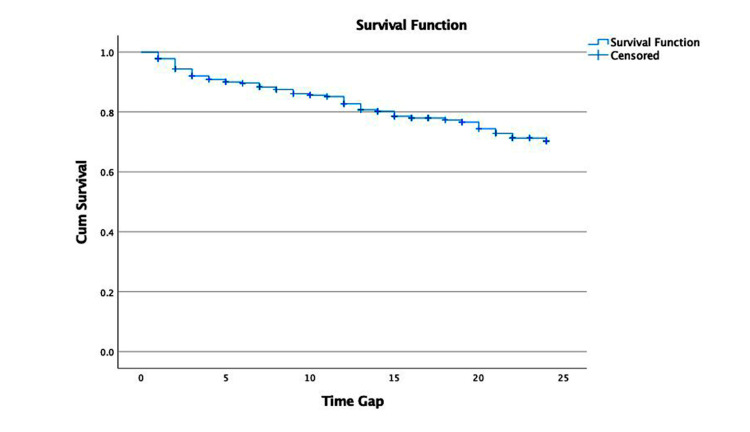
Mean survival among gastric cancer patients.

**Figure 2:**
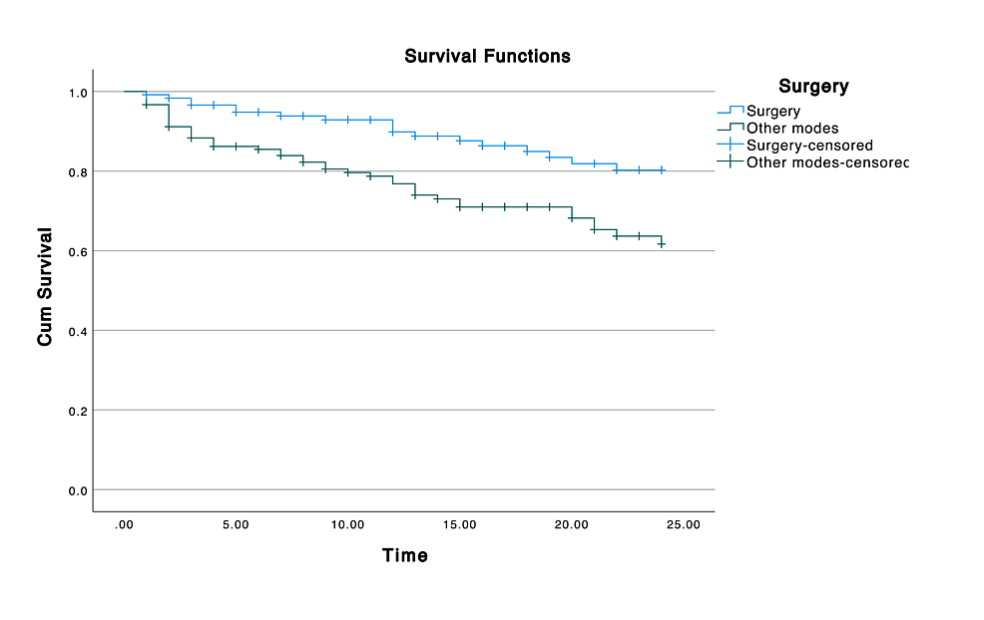
Two-year survival rate according to surgical intervention.

**Figure 3:**
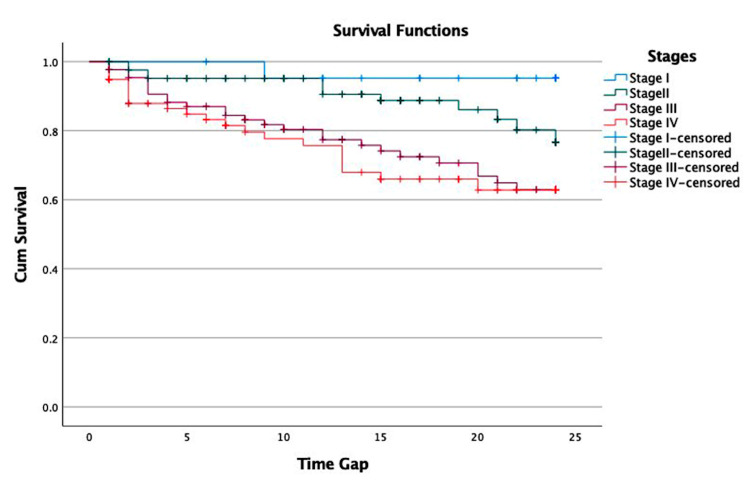
Two-year survival rate according to the stage of gastric cancer.

The mean overall survival (OS) of gastric cancer patients was 20.10 months (95% confidence interval = 19.20-21.03) (Figure 1). The mean survival among patients undergoing surgery was better compared to their counterparts (Figure 2). The mean survival among stage IV patients was the lowest (18.20 months), followed by stage III (19.05 months) patients. Survival among stage I and II patients was significantly higher (p = 0.003) (Figure 3). Two years of mean survival was 72.2% among stage IV gastric cancer patients and 70.5% among stage III patients. Two years of mean survival was higher in stage I (95.8%) and stage II (85.9%) patients.

## Discussion

Different countries have varying rates of gastric cancer incidence and mortality. Although the incidence of gastric cancer has been declining [10], its survival in low- and middle-income countries is still low except for Japan, where mass screening and early detection play a major role [11]. In the state of Odisha, gastric cancer is the second most common among males and fourth among females [12]. However, data on survival are lacking.

The mean two-year OS for gastric cancer patients in our study was 20.097 months, which is lower compared to previous studies [13,14]. An Iranian study reported one-year, three-year, and five-year median survival rates of 66%, 31%, and 21.60% respectively. Although surgery is considered the best treatment in the early stages, due to late diagnosis chemotherapy and radiotherapy did not increase the patients’ survival [7]. A study from Turkey also showed that advanced-stage gastric cancer has a lower survival rate [9]. Most cases of gastric cancers are detected at an advanced stage. In our study, the majority of patients were diagnosed in stages III and IV. Patients who were diagnosed at an advanced stage had higher mortality, for example, stage IV gastric cancer patients had the poorest OS rate than those in other stages. There was a reduced survival of stage III and IV patients compared to stage I and II patients at a statistically significant level.

In our study, the male-to-female ratio was 1.54:1, similar to many other studies, demonstrating that men are more likely than women to be diagnosed with gastric cancer [5,9,15,16]. The lowest male-to-female ratio of 1.2:1 [17] and the highest of 3:1 [5] has been reported in the literature.

The age distribution in the majority of patients with gastric cancer has been in the older (fifth or sixth decade) age group [6]. The average age of male patients in our study was similar (males: 59 ± 12.83 years; females: 54.01 ± 13.41 years) to that reported by Samantaray et al. from the state of Odisha [5]. In our study, most (94.2%) patients had gastric adenocarcinoma, congruent with findings of other studies from India, with gastric adenocarcinoma accounting for 90-95% [6].

In our study, patients who received surgery alone had a lower mortality rate at a statistically significant level compared to other modes of intervention. However, it has been reported that the OS is longer for patients who received surgery with chemotherapy rather than those who received surgery, chemotherapy, and radiotherapy alone [18]. This can be attributed to late diagnosis, as reported by an Iranian cancer institute experience [7].

Gastric cancer patients are often plagued by various symptoms, including abdominal pain, melena (abdominal discomfort), difficulty swallowing, hematemesis (diarrhea), and weight loss. In our study, patients with other GI complaints such as nausea and hiccups had a higher mortality rate than those without other problems.

Our study had several limitations. Being a retrospective study [19,20], the inherent bias (incidence-prevalence bias) cannot be excluded, and because it was a single institution, hospital-based study, generalization to the entire population would be difficult. Although the five-year survival is a standard method of representation, due to a lack of available data, we have only presented two-year survival. Being a retrospective study, more clinically relevant information (such as duration of symptoms, endoscopic and other radiological findings, the extent of surgery, and the type of chemotherapy) and other risk factors could have been presented. However, poor data keeping was a limitation. Most patients were diagnosed either in stage III or IV in our study setting and the reasons could not be explored as this is a record-based study. The lag time between upper GI endoscopy and the diagnosis was not considered in this study, which may underestimate the survival. Further, we have reported data only for two years from a single center using hospital records. However, being a premier oncology unit, it caters to a large population.

## Conclusions

The overall two-year survival rate of gastric cancer patients in our study setting was 20.097 months. A higher survival was observed among those diagnosed with early pathological stages and those who had surgery as an intervention compared to chemotherapy and/or radiotherapy, emphasizing the role of early diagnosis and early surgical intervention in improving the survival of gastric cancer patients.
